# The bright and dark side of extracellular vesicles in the senescence-associated secretory phenotype

**DOI:** 10.1016/j.mad.2020.111263

**Published:** 2020-07

**Authors:** Ryan Wallis, Hannah Mizen, Cleo L. Bishop

**Affiliations:** Centre for Cell Biology and Cutaneous Research, Blizard Institute, Barts and The London School of Medicine and Dentistry, Queen Mary University of London, 4 Newark Street, London E1 2AT, UK

**Keywords:** Ageing, Senescence, Extracellular vesicles, EVs, Inflammation, SASP, miRNA

## Abstract

Senescence is a state of proliferative arrest which has been described as a protective mechanism against the malignant transformation of cells. However, senescent cells have also been demonstrated to accumulate with age and to contribute to a variety of age-related pathologies. These pathological effects have been attributed to the acquisition of an enhanced secretory profile geared towards inflammatory molecules and tissue remodelling agents – known as the senescence-associated secretory phenotype (SASP). Whilst the SASP has long been considered to be comprised predominantly of soluble mediators, growing evidence has recently emerged for the role of extracellular vesicles (EVs) as key players within the secretome of senescent cells. This review is intended to consolidate recent evidence for the roles of senescent cell-derived EVs to both the beneficial (Bright) and detrimental (Dark) effects of the SASP.

## Highlights

• Extracellular vesicles (EVs) are key mediators within the senescence-associated secretory phenotype (SASP).
• Increased EV production has been demonstrated following senescence induction.
• Changes in EVs cargoes including proteins, nucleic acids and lipids have been demonstrated following senescence induction.
• EVs have been demonstrated to contribute to both the beneficial (Bright) and detrimental (Dark) sides of the SASP.

## 1 Introduction

### 1.1 Senescence and the senescence-associated secretory phenotype (SASP)

Senescence is a stable state of proliferative arrest which occurs in cells exposed to a variety of damaging stressors including telomere shortening, DNA-damaging agents, irradiation, hydrogen peroxide (H_2_O_2_), tumour suppressor loss, autophagy impairment and oncogene-expression (Sharpless and Sherr, 2015; Gorgoulis et al., 2019). Senescence is widely considered to be a protective mechanism, which acts to prevent the malignant transformation of cells by instigating a mostly irreversible cell-cycle arrest in response to potentially oncogenic stimuli (Munoz-Espin and Serrano, 2014). A key hallmark of senescence is the acquisition of an enhanced secretory profile through which senescent cells modulate their local microenvironment – the senescence-associated secretory phenotype (SASP) (Coppe et al., 2008; Coppé et al., 2010). One key physiological role of the SASP is the removal of senescent cells, via immune recruitment, following senescence induction (Xue et al., 2007; Kang et al., 2011; Munoz-Espin and Serrano, 2014; Yun et al., 2015). Another, is the contribution of the SASP to optimal wound healing following injury (Demaria et al., 2014; Baker et al., 2016). However, whilst these physiological roles may be considered the “bright” side of the SASP it has also been demonstrated to have “dark” pathological effects during ageing (Coppé et al., 2010). Senescent cells have been demonstrated to accumulate with age (possibly due to immune impairment) and, through the SASP, are implicated in a variety of age-related pathologies including osteoarthritis, atherosclerosis, diabetes, kidney dysfunction, neurodegeneration and vision loss (Baker et al., 2016; Childs et al., 2016; Baar et al., 2017; Ogrodnik et al., 2017; Xu et al., 2018). A key pathogenesis which links these diseases is the maintenance of a persistent state of chronic sterile inflammation (known as inflammageing) to which the SASP has been proposed as a major contributor (Franceschi and Campisi, 2014). Furthermore, depending on the setting, the SASP has been demonstrated to be both pro- and anti- tumorigenic (Bavik et al., 2006; Coppe et al., 2008; Coppé et al., 2010; Acosta et al., 2013). These diverse roles are driven by heterogeneity in the composition of the SASP, which occurs as a result of both cell type and senescence trigger, giving the SASP an intensely context dependent role (Gorgoulis et al., 2019). Furthermore, the SASP has been demonstrated to be a dynamic phenotype, developing during the course of senescence induction (Hoare et al., 2016). These changes are driven by the transcription factors NF-κB and C/EBPβ, which have been identified as key determinants of SASP composition (Acosta et al., 2008; Kuilman et al., 2008; Rodier et al., 2009; Chien et al., 2011). To date, the functions of the SASP have been predominantly attributed to soluble proteins (Coppé et al., 2010). However, emerging evidence suggests extracellular vesicles (EVs) may play a key role in the complex and varied roles of the SASP (Takasugi, 2018).

## 2 Biogenesis and sub-classes of extracellular vesicles (EVs)

Following their discovery in 1983, EVs were primarily thought to be a route for the removal of harmful cellular waste (Pan and Johnstone, 1983; Rashed et al., 2017). However, increasingly, their role in cellular communication is becoming appreciated, particularly since the discovery that EVs can deliver functional RNA to recipient cells (Valadi et al., 2007; Edgar et al., 2016). Generally defined as lipid bilayer bound vesicles secreted by cells, EVs consist of a highly heterogeneous population that differs in size, function and biogenesis. This heterogeneity is reflected in the varied nomenclature that has been used to describe EV sub-types. However, efforts have recently been made to reach consensus when defining EV populations (Lotvall et al., 2014; Théry et al., 2018).

**Fig. 1:**
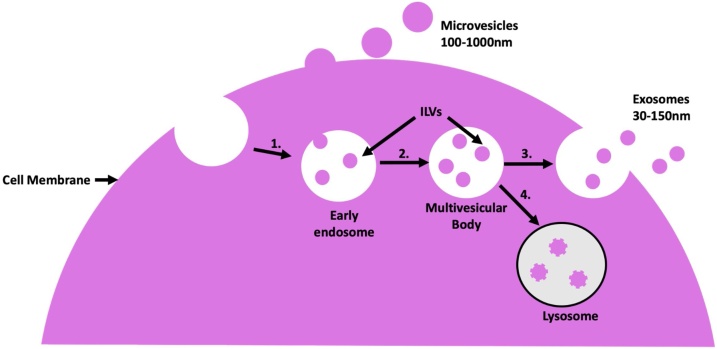
Biogenesis pathway of Extracellular Vesicles (EVs). Microvesicles are formed from outward budding of the plasma membrane of cells encapsulating various cargoes including RNA and proteins from the cytoplasm. Exosome biogenesis pathway: 1. The early endosome repeatedly undergoes inward budding forming intraluminal vesicles (ILVs) using either ESCRT dependent or independent mechanisms. 2. This results in a multivesicular body (MVB). 3. The MVB then fuses with the plasma membrane and ILVs are released into the extracellular space where they classified as exosomes. 4. Alternatively, MVBs fuse with the lysosome allowing ILVs to be broken down.

Exosomes represent the smallest and most widely investigated subset of EVs and are defined by their size (50−150 nm) and endosomal origin (Colombo et al., 2014; Edgar et al., 2016). The budding of endosomal membranes leads to formation of multivesicular bodies (MVBs) containing intraluminal vesicles (ILVs) (Fig. 1) (Gould and Raposo, 2013). The endosomal sorting complex required for transport (ESCRT) is key to this process, mediating the recruitment of cargos, budding and eventual cleavage processes of the endosomal membrane (van Niel et al., 2018). The ESCRT machinery is subdivided into four domains which are utilised in a stepwise manner during MVB formation. Initially, ESCRT-0 and -I subunits, such as TSG101 (tumour susceptibility gene 101), and accessory proteins, such as ALIX (ALG-2-interacting protein X) and syntenin, cluster transmembrane and cytoplasmic cargos at microdomains on the MVB membrane. This is followed by recruitment of ESCRT-II and -III and associated proteins which perform membrane budding and scission to release intraluminal vesicles (ILVs) (Colombo et al., 2013). Components involved in MVB formation, such as TSG101 and ALIX, are considered markers of exosomal biogenesis (Tamai et al., 2010; Baietti et al., 2012).

An ESCRT-independent biogenesis pathway also exists, which may utilise the clustering of cone shaped lipids to trigger spontaneous membrane budding (Tkach and Théry, 2016). This alternative pathway has been demonstrated to produce CD63 enriched ILVs following ESCRT knockdown (Stuffers et al., 2009). CD63 is a tetraspanin which, alongside CD9 and CD81, also features in exosome biogenesis, clustering with other membrane proteins and contributing to the sorting of exosomal cargoes (Buschow et al., 2009; Chairoungdua et al., 2010; Charrin et al., 2014). ILV formation may also be driven through the production of ceramide by neutral sphingomyelinase, which facilitates the required endosomal membrane curvature (van Niel et al., 2018). Importantly, this process may be perturbed through use of the inhibitor GW4869, which has proven a useful experimental tool (Trajkovic et al., 2008). Whilst the process of ILV biogenesis is complex and not yet fully elucidated, ILV formation leads to classification of endosomes as MVBs. MVBs have several possible fates, one of which is fusion with lysosomes for enzymatic degradation and, indeed, in cells with impaired lysosomal function, exosome production has been demonstrated to increase (Eitan et al., 2016; Schorey and Harding, 2016) — potentially acting as an alternative mechanism through which to dispose of harmful or defective proteins. For ILVs to be released from a cell, thus becoming exosomes, the MVB must avoid trafficking to the lysosome in favour of the plasma membrane. The cargo and composition of ILVs plays a key role in their eventual trafficking fate, with the formation of a complex between ALIX, syntenin and syndecans (heparin sulphate proteoglycans) leading exclusively to the release of ILVs as exosomes (Baietti et al., 2012). Furthermore, differential trafficking fates have also been attributed to the cholesterol levels within MVBs, with ILVs containing high cholesterol levels preferentially released as exosomes (Möbius et al., 2003).

The RAB superfamily of small GTPases are also crucial for MVB trafficking to the plasma membrane, with the ubiquitination status of RAB7 believed to be essential in determining lysosome or plasma membrane trafficking (Baietti et al., 2012; Song et al., 2016). RAB27a and RAB27b are also key mediators of exosome release, regulating MVB fusion with the plasma membrane (Ostrowski et al., 2010). Other RAB family members (including RAB11, RAB35 and their effectors) have also been reported to perform similar roles in cells that do not constitutively express RAB27. A comprehensive review of the role of RABs in exosome biogenesis may be found here (Blanc and Vidal, 2018). Once secreted, exosomes can interact with recipient cells through numerous mechanisms, including endocytosis and direct membrane fusion, leading to the intracellular delivery of luminal EV cargoes (Thery et al., 2009; Mulcahy et al., 2014; van Niel et al., 2018).

Other EV sub-populations of note include microvesicles, which bud directly from the plasma membrane (Fig. 1). Whilst not as extensively studied as exosomes, it has been shown that the machinery which drives the biogenesis of exosomes, including ESCRT proteins and tetraspanins, are also crucial for the formation of microvesicles. Due to this, it is difficult to confidently identify isolated EVs as exosomes (Gould and Raposo, 2013; Lotvall et al., 2014; Witwer and Théry, 2019). Therefore, we have decided to use the general term extracellular vesicles (EVs) rather than a specific sub-type of vesicle throughout this review (Witwer et al., 2013).

## 3 Changes in extracellular vesicle production and secretion in senescence

Whilst the enhanced secretome of senescent cells has been extensively investigated, changes in EV secretion by senescent cells has only recently received significant research interest (Coppé et al., 2010; Takasugi, 2018). Lehmann et al. (2008) were the first to investigate the association between EVs and senescence, demonstrating that irradiation-induced senescence caused an increase in EV production from human prostate cancer cells (Lehmann et al., 2008). This finding, supported previous work conducted by Yu and colleagues (2006), who demonstrated that an increase in EV production from irradiated non–small cell lung cancer cells was dependent on the p53 targets tumour suppressor-activated pathway 6 (TSAP6) and vacuolar protein sorting-associated protein 32 (VPS32; a key part of the ESCRT complex), although the investigators did not look at senescence specifically (Yu et al., 2006). Furthermore, Lespagnol et al. (2008) demonstrated a p53-dependent increase in EV secretion following DNA damage, which was abrogated in TSAP6-null mice (Lespagnol et al., 2008). Interestingly, p53 was also implicated in the upregulation of RAB27b (a key component of EV secretory pathways) in senescent human SVts8 fibroblasts (Fujii et al., 2006). Following these early investigations, it has recently been demonstrated that normal TIG-3 human fibroblasts produce a greater number of EVs in response to senescence inducing-stimuli including replicative senescence, oncogenic RAS expression and the chemotherapeutic doxorubicin (Takasugi et al., 2017). Subsequent investigations have demonstrated an increase in EV production in models of therapy-induced (Kavanagh et al., 2017), H_2_O_2_-induced (Terlecki-Zaniewicz et al., 2018), irradiation-induced (Basisty et al., 2020), oncogene-induced (Borghesan et al., 2019), and replicative senescence (Mensà et al., 2020; Riquelme et al., 2020). Therefore, it appears that, much like the soluble secretome, EV production increases in multiple cell types following a variety of different senescence-inducing stimuli.

The mechanisms which underpin the increased production of EVs in senescence have not been extensively studied. However, recent work by Choi, Kil and Cho, demonstrated that senescent dermal fibroblasts upregulate neutral sphingomyelinase (a key enzyme in ILV biogenesis) which led to a reduction in EV secretion when inhibited (van Niel et al., 2018; Choi et al., 2020). Furthermore, the investigators proposed a link between the dysfunctional lysosomal activity of senescent cells and the increased rate of EV production, as artificially lowering lysosomal pH in senescent fibroblasts perturbed EV production, whilst increasing pH in proliferating fibroblasts increased EV production (Choi et al., 2020). Together, this research implicates dysfunctional lysosomal activity as a major factor affecting the rate of EV production by senescent cells.

## 4 Changes in extracellular vesicle cargo in senescence

EV cargo is derived from the cell of origin, and, as such, can provide information about the composition of that cell, at the time of EV production (Saleem and Abdel-Mageed, 2015; McBride et al., 2017). However, as described above, EVs are also enriched for a variety of cargoes, some of which are involved in their biogenesis (van Niel et al., 2018). Nevertheless, as EVs derive their cargo directly from a producing cell, changes in cellular content following senescence induction may alter the composition and luminal cargo of EVs derived from those cells. The changes in EV composition have now been studied extensively in a variety of cell types, following a variety of senescence triggers (Kadota et al., 2018; Takasugi, 2018). These are discussed below in the context of protein, nucleic acid and lipid constituents.

### 4.1 Proteins

Soluble proteins have been the most widely investigated factors within the SASP with constituents including inflammatory cytokines (e.g. interleukin-1, (IL-1), IL-6), chemokines (e.g. IL-8), growth factors (e.g. epidermal growth factor (EGF)) and proteases (e.g. matrix metalloproteinase-1 (MMP1)) (Coppé et al., 2010). However, recently, characterisation of EVs and their functional protein cargoes have garnered greater attention and revealed a distinct role as regulatory units (Takasugi, 2018; Basisty et al., 2020). Takasugi et al, (2017) performed proteomic analysis of EVs following senescence induction using doxorubicin (Takasugi et al., 2017). The investigators demonstrated that EVs isolated from senescent cells promoted the proliferation of several types of cancer cell, whilst senescent cell derived conditioned media depleted of EVs attenuated a pro-proliferative effect on MCF-7 breast cancer cells. The tyrosine kinase receptor ephrin receptor A2 (EphA2) was identified as being upregulated in EVs from doxorubicin-induced senescent cells and established as a key functional cargo for the pro-proliferative effects of the EVs. Strikingly, this role of EV EphA2 could not be recapitulated using the soluble form of the receptor. This phenomenon, which suggests that EV derived cargo may be more potent than the soluble form of the same protein, has previously been reported for the soluble factors interferon gamma (IFN-γ) (Cossetti et al., 2014) and transforming growth factor beta (TGF-β) (Webber et al., 2015). This not only emphasises the distinction between EVs and the soluble SASP but also raises the intriguing possibility that EVs may play a role in enhancing the signalling of soluble factors.

EVs have also been derived from cancer cells which have undergone therapy-induced senescence (TIS) (Kavanagh et al., 2017). Investigators identified changes in key proteins involved in cell proliferation, ATP depletion, apoptosis and the SASP. It was suggested that EVs may represent a route through which TIS cancer cells dispose of these proteins and thus contribute to cancer cell survival and resistance to chemotherapy.

One of the key roles of the SASP is the ability to confer paracrine senescence on neighbouring cells (Acosta et al., 2013). A recent study by Borghesan et al., demonstrated that EVs derived from oncogenic RAS-induced fibroblasts can contribute to this effect (Borghesan et al., 2019). Inhibition of EV production was sufficient to attenuate the paracrine senescence effect of the SASP. Furthermore, the investigators demonstrated that EV paracrine senescence was partially mediated through interferon-induced transmembrane protein 3 (IFTM3). These findings collectively highlight the potential of EVs as mediators of cellular communication between senescent cells and the local microenvironment.

Recently, efforts have been made to fully characterise the secretome of senescent cells by generating a so called “Atlas” of SASP and EV factors in different cell types and senescence models (Basisty et al., 2020). Investigators demonstrated little overlap in protein composition between these two components of the senescent secretome. Therefore, from both a functional and compositional perspective, EVs appear to have a distinct role as regulatory mediators beyond the traditional soluble factors which have previously been considered as the main players within the SASP.

### 4.2 Nucleic acids

Functional transfer of nuclei acids between cells via EVs was first demonstrated in 2007 when Valadi et al. demonstrated RNA transfer from mouse to human mast cells via EVs (Valadi et al., 2007). This finding led to an increase in research interest surrounding EVs as a mechanism for cellular communication, with miRNA in senescent cell derived EVs first being investigated in 2015 (Weiner-Gorzel et al., 2015). The potential of EVs to deliver nucleic acid cargoes, such as upregulated miRNAs, from senescent cells, to the cytoplasm of proliferative cells, adds an additional facet to the SASP, as recently demonstrated with an assortment of miRNAs triggering senescence induction in previously proliferating cells (Overhoff et al., 2014; Luo et al., 2015; Xu et al., 2019). miRNAs are the most widely investigated nucleic acid cargo of EVs and are, therefore, the most extensively discussed in this review. However, a variety of additional nucleic acids including DNA, Telomeric repeat-containing RNA (TERRA) and mitochondrial DNA (mtDNA) have been identified in senescent cell derived EVs.

#### 4.2.1 miRNA

Early research investigating the association between senescence, EVs and miRNAs demonstrated that senescence induction via overexpression of miR-433 in A2780 ovarian cancer cells also resulted in the packaging of miR-433 into EVs. Through this miRNA cargo, the EVs were implicated as potential contributors to a paracrine senescence response in recipient cells (Weiner-Gorzel et al., 2015). Recently, investigations by Fulzele *et al*., demonstrated that the serum of aged mice contained EVs with increased levels of miR-34a. Upregulation of miR-34a was also detected in muscle derived EVs present in the serum of aged mice and in EVs derived from human myotubes undergoing oxidative stress from H_2_O_2_. Furthermore, EVs isolated from H_2_O_2_ treated mouse myoblasts, or mouse myoblasts overexpressing miR-34a, were found to contain increased levels of miR-34a. The latter two of these EV populations caused a paracrine senescence effect when cultured with bone marrow stem cells *in vitro,* compared to EVs from untreated mouse myoblasts. Of note, miR-34a has been shown by others to indirectly regulate p53 through the degradation of the p53 suppressor Sirtuin 1 (SIRT1), which can result in senescence induction (Yamakuchi and Lowenstein, 2009). Fulzele et al. further demonstrated that EVs could be detected in multiple tissues, including the fore- and hind-limb, following tail-vein injection of EVs derived from miR-34a overexpressing mouse myoblasts into healthy mice. Additionally, bone marrow cells isolated from the limbs of untreated mice were cultured *ex vivo* with either EVs from mouse myoblasts overexpressing miR-34a or EVs isolated from control cells. Finally, the authors determined that treatment with EVs from mouse myoblasts overexpressing miR-34a resulted in a reduction in SIRT1 at both the mRNA and protein level in bone marrow cells, although the functional consequences of this reduction were not explored (Fulzele et al., 2019). In combination with SIRT1, DNA methyltransferase 1 (DNMT1) acts to ensure genomic integrity and exert pro-longevity effects. Mensa et. al., explored this by screening replicatively senescent human umbilical vein endothelial cells (HUVECs) and their small EVs for miRNAs that target SIRT1 and/ or DNMT1. Several miRNAs were identified including miR-21-5p and miR-217, which the authors demonstrated were upregulated in replicatively senescent human aortic endothelial cells (HAECs) and their EVs. This was also observed in a model of drug-induced senescence established in both HUVECs and HAECs. Senescent EVs were able to transfer miR-21-5p and miR-217 to recipient cells, which resulted in reduced expression of both SIRT1 and DNMT1, leading to a decrease in cell proliferation and an increase in senescent cell markers, including p16, IL-6 and IL-8 mRNA (Mensà et al., 2020). Therefore, EV derived miRNA cargo from aged cells may constitute a potential “dark” pathological role of EVs by facilitating the propagation of senescence between tissues, raising the possibility that similar interactions could be driven by a diverse set of miRNAs and cell types in a context dependent manner.

Wound healing has often been reported as one of the “bright” beneficial effects of the SASP (Demaria et al., 2014) and, interestingly, senescent human dermal fibroblast derived EVs containing miR-23a-3p have been shown to play a role in wound healing through the transfer of this miRNA to non-senescent keratinocytes, resulting in more efficient wound healing (Terlecki-Zaniewicz et al., 2019). The miRNA profile of EVs derived from H_2_O_2_-induced senescent human fibroblasts was altered when compared to EVs from quiescent fibroblasts and, intriguingly, developed with time after senescence induction. Furthermore, the authors identified miRNAs that are specifically packaged into EVs for secretion from the senescent cell, including miR-15b-5p and miR-29c-3p, and miRNAs that are specifically retained by senescent cells, such as miR-409-5p and miR-323a-3p. The top 20 most secreted miRNAs were predicted to target transcription factors that are known pro-apoptotic mediators, suggesting a role for EV-packaged miRNAs as anti-apoptotic members of the SASP. The authors confirmed this potential role by demonstrating that EVs from H_2_O_2_-induced senescent human fibroblasts reduced apoptosis in recipient fibroblast cells undergoing acute stress from high H_2_O_2_ levels, compared to EVs from quiescent control fibroblasts (Terlecki-Zaniewicz et al., 2018). Therefore, EV carried miRNAs represent underappreciated yet important players within both the “bright” and “dark” sides of the SASP.

#### 4.2.2 Telomeric repeat-containing RNA (TERRA) and telomeric DNA

Telomere shortening – a key facet of replicative senescence – increases the expression of Telomeric repeat-containing RNA (TERRA) within the cell cytoplasm (Cusanelli et al., 2013; Takasugi, 2018); TERRA has been identified in EVs and has been demonstrated to induce inflammatory gene expression in recipient cells (Wang et al., 2015b; Wang and Lieberman, 2016). However, a link to increased TERRA in senescent EVs has not yet been established. Contrasting the effects of TERRA, cytoplasmic telomeric DNA has been shown to have an anti-inflammatory role, as reviewed previously (Bonafè et al., 2020). The authors speculate that telomeric DNA fragments could be packaged into EVs and exert anti-inflammatory benefits on recipient cells in order to delay age related pathologies.

#### 4.2.3 DNA

The presence of cytoplasmic chromatin DNA fragments (CCFs) within senescent cells has been recently established (Adams et al., 2013), with CCFs potentially acting as indirect regulators of the SASP through the cGAS-STING pathway. In brief, cGAS directly binds CCFs and subsequently synthesises cyclic GMP-AMP (cGAMP), which in turn activates the endoplasmic reticulum adaptor STING, leading to activation of crucial SASP transcription factors including NF-κB. (Dou et al., 2017; Glück et al., 2017). Chromosomal DNA secretion from cells within EVs has been demonstrated to increase with senescence, and appears to maintain cellular homeostasis by removing potentially harmful aberrant DNA, thus avoiding a ROS dependent DNA damage response (Takasugi et al., 2017). This principle was validated by the inhibition of EV section through both siRNA knockdown of ALIX or RAB27a, or through use of the EV secretion inhibitor GW4869, which led to an increased expression of the DNA damage marker γ-H2AX and phosphorylation of key DNA damage response kinases – ATM and ATR (Takahashi et al., 2017; Takasugi et al., 2017). Inhibition of CCFs excretion in EVs also resulted in cells becoming prone to apoptosis. This pathway implicates EV secretion as a homeostatic mechanism, returning to the concept of “waste disposal” first suggested decades earlier (Johnstone, 1992). Interestingly, CCF containing vesicles could elicit a DNA damage response in recipient TIG-3 fibroblasts suggesting that, whilst this mechanism may be beneficial to the producing cell, it may have detrimental consequences in the surrounding microenvironment (Takahashi et al., 2017). This is reminiscent of the SASP as a whole and suggests that senescent cell derived EVs and their cargo may contribute to both sides of the bright and dark dichotomy of the SASP.

#### 4.2.4 Mitochondrial DNA (mtDNA)

Along with cellular senescence, mitochondrial dysfunction has been proposed as one of nine key “pillars” of ageing (López-Otín et al., 2013). Oxidative stress is a key trigger of senescence and excessive reactive oxygen species (ROS) production has been implicated in the protein damage considered to be a hallmark of senescence and ageing (Kaushik and Cuervo, 2015; Höhn et al., 2017). Accumulation of aberrant cellular products due to mitochondrial dysfunction underpins the “garbage catastrophe” theory of ageing, whereby the production of cellular waste products outstrips disposal or recycling mechanisms, thus leading to impairment of homeostasis (Terman et al., 2010; Baixauli et al., 2014; Picca et al., 2019). As discussed above, EVs have long been considered a potential route for cellular waste disposal and it has been proposed that EVs may serve as a reserve mechanism to meet demand where excessive waste production overcomes lysosomal capacity, particularly during oxidative stress (Soubannier et al., 2012; Picca et al., 2019). This is supported by the observable increase in EV secretion following H_2_O_2_ induced senescence (Terlecki-Zaniewicz et al., 2018). Furthermore, mitochondrial DNA (mtDNA) has been identified in EVs isolated from astrocytes (Guescini and Genedani, 2010) and myoblasts (Guescini and Guidolin, 2010) suggesting that mitochondrial components may be removed via a vesicular route. Supporting this concept is evidence that oxidative stress can cause astrocytes (Hayakawa et al., 2016) and mesenchymal stem cells (Phinney et al., 2015) to release EVs containing mitochondrial particles (Picca et al., 2019). Therefore, the reprogramming of cellular metabolism in response to oxidative stress-induced senescence may involve the use of EVs to dispose of aberrant mitochondrial components. Parallels to this may be drawn in therapy resistant metastatic breast cancer cells which were demonstrated to contain a complete mitochondrial genome (Soubannier et al., 2012). Furthermore, these cells were able to produce EVs containing mtDNA in response to oxidative stress, contributing to therapeutic resistance (Soubannier et al., 2012). The consequences of these EVs to cells in the surrounding microenvironment is not known and is worthy of further investigation. Overall, EVs represent an underexplored dimension to the metabolic reprogramming which occurs following senescence induction.

### 4.3 Lipids

A relatively underexplored facet of senescent cell derived EVs is their lipid profile. Several recent publications have identified changes in the lipid profiles of oncogene-induced senescent fibroblast derived EVs. Enrichment was demonstrated in hydroxylated sphingomyelin, lyso- and ether-linked phospholipids, when compared to the producing cell, indicating that the lipid composition of EVs may differ from the parental cells more than previously appreciated (Buratta et al., 2017). Furthermore, a subsequent study identified that EVs from oncogene-induced senescent fibroblasts showed increased levels of monounsaturated and decreased level of saturated fatty acids, as compared to control cells (Sagini et al., 2018). Intriguingly, EVs from bone-derived mesenchymal stem cells have been demonstrated to contribute to paracrine senescence when loaded with very long chain C24:1 ceramide (Khayrullin et al., 2019). The lipid composition of senescent cell derived EVs, therefore, potentially represents an underexplored mechanism through which senescent cells contribute to the paracrine effects of the SASP.

## 5 Role of extracellular vesicles in ageing

### 5.1 Changes in EV production with aging

Senescent cells have previously been demonstrated to accumulate with age and at sites of age-related diseases (Nielsen et al., 1999; Krishnamurthy et al., 2004, 2006). Given that, as described above, senescent cells have an increased rate of EV secretion, it might be predicted that EV production would increase with ageing. However, contradictory data has emerged regarding the change in EV production with age. An initial study concluded that, although the soluble SASP component IL-6 increased in aged volunteers, EV concentration did not alter (Alberro et al., 2016). Another suggested that fewer EVs could be isolated from the plasma samples of elderly patients. However, the authors also noted that circulating EVs were increased in individuals that smoked or had higher a body mass index (BMI), both of which are correlated with higher levels of senescent cells in tissues (Nyunoya et al., 2006; Eitan et al., 2017). By contrast, circulating EVs have also been demonstrated to increase in aged subjects (Im et al., 2018) and in mouse models of normal ageing (Alibhai et al., 2020; Qureshi et al., 2020). Aged chondrocytes isolated from arthritic cartilage have also been shown to produce an increased number of EVs. (Jeon et al., 2019). One possible explanation for these conflicting data is a reported increase in clearance of EVs in ageing, which may mask increased production in certain settings (Eitan et al., 2017). Another consideration, is the use of different methods of EV characterisation, with one study demonstrating that whilst the number of circulating particles detected by nanoparticle tracking analysis (NTA) was higher in young (3 month) mice, EV markers were enriched in old (18–21 month) mice as determined by western blot (Alibhai et al., 2020). This demonstrates the importance of utilising multiple analysis approaches when characterising EVs (Lötvall et al., 2014). Further complexity also comes from the suggestion that EV concentration increases with certain age-related diseases (Tan et al., 2017). Finally, EV concentration could be affected by confounding factors including medication used in the treatment of age-related pathologies, as it has been shown that the regular use of aspirin in elderly patients reduced the number of platelet derived EVs without altering the overall number of circulating EVs (Goetzl et al., 2016).

### 5.2 Changes in EV cargo with age

Whilst the level of EV production in age remains an open question, it is clear from numerous studies that EV composition changes with age. miR-31 has been demonstrated to increase and Galectin-3 determined to decrease, in EVs isolated from elderly patients. These changes resulted in both the impairment of osteogenic differentiation of mesenchymal stem cells (MSCs) and compromised tissue regeneration (Weilner and Keider, 2016; Weilner and Schraml, 2016). Furthermore, EVs from the bone marrow of aged mice have also been demonstrated to reduce osteogenic differentiation, in this case through increased miR-183-5p expression, leading to a reduction in bone mineral density and bone function (Davis et al., 2017). The mitochondrial content of immune cell derived EVs was also recently shown to decline with age (Picca et al., 2019; Zhang et al., 2020). Macrophage-derived serum EVs from aged (>65 years) individuals were also shown to contain significantly higher levels of IL-6 and IL-12 mRNA than matched EVs from young (21–45 years) volunteers (Mitsuhashi et al., 2013). The miRNA cargo of EVs has also been implicated as a potential biomarker of pathological aging. Ipson et al., identified 8 miRNAs that are increased in EVs from frail older individuals compared to both robust older and young patients (Ipson et al., 2018). Furthermore, expression of miR-21 has been previously recognised to increase in pathological ageing (Olivieri et al., 2012) and was recently demonstrated to be one of several miRNAs that were upregulated in EVs in both aged mice and an irradiation induced global model of senescence (Alibhai et al., 2020). miR-21-5p has also recently been proposed as a potential marker of senescence and inflammageing (Mensà et al., 2020).

Changes in EV cargoes have also been described in pathologies associated with age. EVs from senescent coronary artery endothelial cells and from patients with acute coronary artery syndrome-induced premature endothelial dysfunction have been demonstrated to propagate a paracrine senescence phenotype (Abbas et al., 2017). Aortic smooth muscle cell calcification may also be facilitated by EVs from senescent endothelial cells, and from the plasma of elderly patients, representing another pathology closely linked to age (Alique et al., 2018). EVs isolated from senescent chondrocytes from patients with osteoarthritis (OA) were also able to induce a paracrine senescence effect in proliferative chondrocytes and inhibited cartilage formation, indicating a role in disease severity (Jeon et al., 2019). Senolytic clearance of senescent cells reduced EV production in OA patients and also altered miRNA cargo composition. Senolytic treatment in aged mice induced primarily immunological changes in EV proteins, and age-related synovial EVs were demonstrated to transfer arthritic disease to young animals, demonstrating a pathological role in OA (Jeon et al., 2019). EVs have also been implicated in the distribution of damaged proteins in neurodegenerative conditions including Alzheimer’s, Huntington’s and Parkinson’s disease (Soria et al., 2017; D’Anca et al., 2019). Furthermore, EV encapsulated miR-21-5p was also found to be upregulated in a range of patients with age-related diseases compared to healthy individuals of the same age or healthy young individuals (Olivieri et al., 2017). Therefore, changes in EV composition have been identified throughout both age and age-related diseases (Urbanelli et al., 2016; Kadota et al., 2018).

### 5.3 Use of EVs as theraputics in age-related pathologies

EVs are emerging as attractive novel therapeutic agents in a variety of settings and have, to date, proceeded as far as phase II clinical trials (Besse et al., 2016). Strikingly, extracellular nicotinamide phosphoribosyl transferase (eNAMPT) containing EVs were recently demonstrated to extend the health-span and increase the physical activity of elderly mice (Yoshida et al., 2019). A variety of different other therapeutic approaches have also been proposed, including the replacement of cell-based therapies with EV treatments and the use of EVs as drug delivery vectors (Ha et al., 2016; Boulestreau et al., 2020). Of particular interest is the use of EVs as a means of RNA interference technology, through loading with a miRNA or siRNA “payload” which could then be delivered to a recipient cell (Hagiwara et al., 2014). EVs present significant advantages over other more established delivery systems, such as liposomes, due to a low toxicity, presence of membrane proteins and a lack of immunogenic effects (Lener et al., 2015; Ohno et al., 2016). Furthermore, efforts have been made to develop EVs which are specifically targeted to cells or tissues through the display of antibody fragments or functional peptides (Ohno et al., 2013; Longatti et al., 2018; Tian et al., 2018). This has the potential to maximise efficacy whilst limiting off-target effects (Ha et al., 2016). Here, we discuss the use of EV based theraeptuics in ageing.

The loss of tissue regeneration represents a universal characteristic of ageing (Gorgoulis et al., 2019). This is driven by a process of stem cell exhaustion which has been proposed (along with senescence) as one of the hallmarks of ageing (López-Otín et al., 2013). Stem cell based therapies have an established regenerative potential within the tissue microenvironment but are limited by adverse effects such as tumour formation (Ma et al., 2020). Harnessing the secretome of stem cells has been proposed as an alternative therapeutic approach which may overcome this shortcoming and utilising stem cell derived EVs (SC-EVs) has become the subject of significant research interest (Boulestreau et al., 2020). SC-EVs have shown promising results in a number of settings, including the regeneration of liver tissue from a fibrotic state in mice (Li et al., 2013; Hyun et al., 2015), improvement of functional recovery after stroke in rats (Xin et al., 2013), restriction of pulmonary hypertension under hypoxic conditions in mice (Lee et al., 2012), protection of cardiomyocytes from apoptosis in ischemic myocardium (Wang et al., 2015a) and acceleration of cutaneous wound healing (Golchin et al., 2018; Chen et al., 2019). SC-EVs have also been modified with functional peptides to target EVs to specific tissues, of particular note is the use of rabies viral glycoprotein (RVG) tagged SC-EVs to target the central nervous system, for the functional improvement of mice in a model of Alzheimer’s disease (Cui et al., 2019). In other studies, RVG tagged EVs have demonstrated the functional delivery of siRNAs to the brain in several models of neurodegenerative disease, thus highlighting the potential of EVs as nucleic acid delivery vectors (Alvarez-Erviti et al., 2011; Cooper et al., 2014). Following these promising preclinical models the International Society for Extracellular Vesicles (ISEV) have released a positional paper for consideration as EV based therapies move into clinical trials (Lener et al., 2015).

Finally, whilst not a true EV based approach, Muñoz-Espín et al., have developed a synthetic nanoparticle based technology which mirrors many of the advantages of EV based therapeutics (Muñoz-Espín et al., 2018). Here, 100 nm silica beads are coated with a layer of galacto-oligosaccharides (GalNP) and upon uptake by recipient cells, these beads are trafficked to the lysosome and subsequently released by exocytosis. However, in senescent cells, high levels of lysosomal β-galactosidase activity digest the GalNP coating leading to a release of any luminal cargo. Investigators used these beads to selectively clear senescent cells through use of chemotherapeutic cargos *in vivo* whilst also reducing the toxicities typically associated with those agents (Muñoz-Espín et al., 2018). Together, these studies highlight the exciting potential of both biological and synthetic nanoparticle based therapeutics in ageing.

## 6 Conclusion

**Fig. 2:**
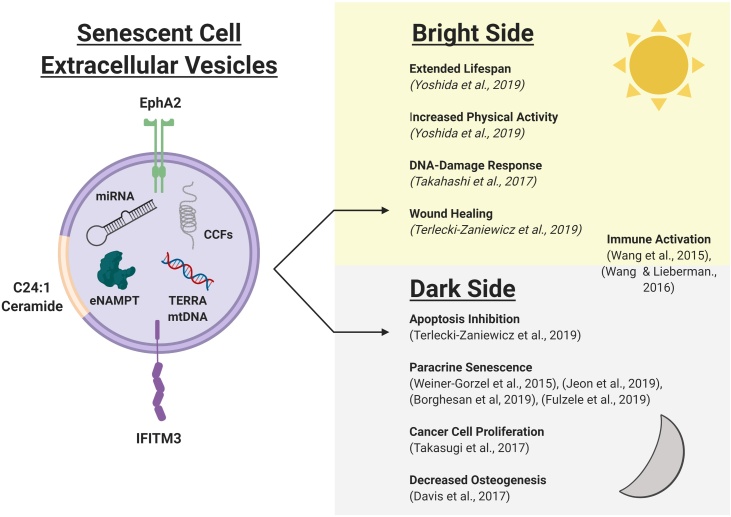
The Bright and Dark Side of Extracellular Vesicles in Senescence and Ageing. The senescence associated secretory phenotype (SASP) has been demonstrated to have beneficial (Bright, yellow) and detrimental (Dark, grey) roles within both normal homeostatic processes and pathology. These diverse and varied effects are driven by heterogeneity in the composition of the SASP, which is influenced by factors including cell type, senescence trigger and disease state. The SASP is also a variable phenotype, developing over the course of senescence induction, leading to significant alterations in constituent factors. Extracellular vesicles (EVs, pink) have recently emerged as key mediators within the SASP and have also been demonstrated to have a wide range of roles in senescence and ageing. As with the more traditional “soluble” SASP, this vesicular secretome also mediates a heterogeneous set of effects that may be considered “bright” or “dark” depending on the specific cellular context. This is driven by a diverse set of EV cargos including proteins, nucleic acids and lipids, which underpin the functional roles of EVs within senescence and ageing. However, as emerging players within the SASP, further work is required to elucidate the contribution of EVs to both the homeostatic and pathological effects of senescent cells. In particular, advancements in isolation and characterisation methodologies will likely lead to the establishment of further heterogeneity in EV sub-populations, adding further complexity to the role of these vesicles within the secretome of senescent cells. EphA2 (Ephrin-A2), C24:1 ceramide (Long chain C24:1 ceramide), IFITM3 (Interferon-induced transmembrane protein 3), TERRA (Telomeric repeat-containing RNA), CCFs (Cytoplasmic chromatin fragments), miRNAs (microRNAs), mtDNA (Mitochondrial DNA) eNAMPT (extracellular nicotinamide phosphoribosyl transferase) Figure created with BioRender. (For interpretation of the references to colour in this figure legend, the reader is referred to the web version of this article).

The SASP is a key hallmark of senescence with both beneficial and deleterious effects in homeostasis and pathology. These numerous and varied roles have thus far predominantly focused on the soluble factors produced including cytokines, chemokines, growth factors and tissue remodelling agents (Coppe et al., 2008; Coppé et al., 2010). However, more recent studies have begun to highlight the role of extracellular vesicles within the SASP (Takasugi, 2018). EVs represent a mechanism through which a wide range of different cargoes may engage in intercellular signalling and, in particular, brings the possibility of the functional transfer of nucleic acids between senescent cells and their microenvironment (Terlecki-Zaniewicz et al., 2018; Jeon et al., 2019). As with the soluble SASP, the role of EVs in senescence and ageing appears to have both beneficial and detrimental effects depending on the cellular context. These are outlined in Fig. 2. However, despite these recent advances, the senescence field must now reflect on the importance of research methodology when investigating EVs, an issue which has recently become the subject of focus from the International Society for Extracellular Vesicles (ISEV) (Lötvall et al., 2014; Théry et al., 2018). In particular, care needs to be employed in separating the soluble and vesicular fractions of the SASP, given the propensity for co-isolation of soluble proteins with EVs when employing the widely used ultracentrifugation method of isolation (Foers et al., 2018). Therefore, in order to confidently attribute effects to EVs derived from senescent cells, efforts must be made to maximise isolation rigor and stringency. Nevertheless, EV production and composition have been extensively demonstrated to change in multiple models of senescence. Whilst more work is required to fully elucidate their role in age and age-related pathologies, EVs represent an alternate method for senescent cells to modulate their local micro-environment beyond the traditional, soluble SASP.
